# Off for Europe

**Published:** 1881-08

**Authors:** 


### OFF FOR EUROPE.

On Saturday, July 16, about thirty of the members of our profession sailed from New York to attend the International Medical Congress to be convened in London on the 3d of this month. The most of these gentlemen are members of the American Dental Association, and are representative men, and can be depended on to maintain the honor and high standard of the profession in America.

Among the number we note Dr. Wm. H Atkinson, of New York; J. Taft, of Cincinnati; Shephard, of Boston; Webb, of Lancaster, Pa.; McKellops, of St. Louis; George Field, of Detroit; Watling, of Michigan University, and Butler, of Cleveland. We venture to say that a party capable of imbibing a greater amount of enjoyment never stepped aboard ship, and that the boys may all have a good time and a safe voyage is our hearty wish.

